# Coralyne Radiosensitizes A549 Cells by Upregulation of CDKN1A Expression to Attenuate Radiation Induced G2/M Block of the Cell Cycle

**DOI:** 10.3390/ijms22115791

**Published:** 2021-05-28

**Authors:** Aneta Węgierek-Ciuk, Michał Arabski, Karol Ciepluch, Kamil Brzóska, Halina Lisowska, Malwina Czerwińska, Tomasz Stępkowski, Krzysztof Lis, Anna Lankoff

**Affiliations:** 1 Institute of Biology, Jan Kochanowski University, Uniwersytecka 7, 25-406 Kielce, Poland; arabski@ujk.edu.pl (M.A.); kciepluch@ujk.edu.pl (K.C.); halina.lisowska@ujk.edu.pl (H.L.); alankoff@gmail.com (A.L.); 2 Centre for Radiobiology and Biological Dosimetry, Institute of Nuclear Chemistry and Technology, Dorodna 16, 03-195 Warsaw, Poland; k.brzoska@ichtj.waw.pl (K.B.); m.wasilewska@ichtj.waw.pl (M.C.); t.stepkowski@cent.uw.edu.pl (T.S.); 3 Remedy International Research Agenda Unit, Centre of New Technologies, University of Warsaw, S. Banacha 2c, 02-097 Warsaw, Poland; 4 Holy Cross Cancer Center, Artwinskiego 3, 25-734 Kielce, Poland; Krzysztof.Lis@onkol.kielce.pl

**Keywords:** coralyne, cellular uptake, cell cycle progression, gene and protein expression, cytoskeletal alterations, cell death

## Abstract

Coralyne is a synthetic analog of berberine related to protoberberine-isoquinoline alkaloids. Isoquinoline derivatives and analogs are renowned as potent radiosensitizers with potential medical application. In the present study, we investigated the effect of coralyne on the cell death, cytoskeletal changes and cell cycle progression of irradiated A549 cells. A clonogenic assay revealed that coralyne pretreatment decreased the viability of A549 cells in a time- and dose-dependent manner. Moreover, exposure to coralyne and ionizing radiation (IR) markedly altered the filamentous actin cytoskeletal architecture and integrin-β binding sites of A549 cells. Treatment with 1–25 µM coralyne in combination with 2 Gy of IR significantly reduced the percentage of cells in G2/M phase compared with 2 Gy IR alone. These results indicate that coralyne is a potent radiosensitizing agent that may find an application in medicine.

## 1. Introduction

**Figure 1:**
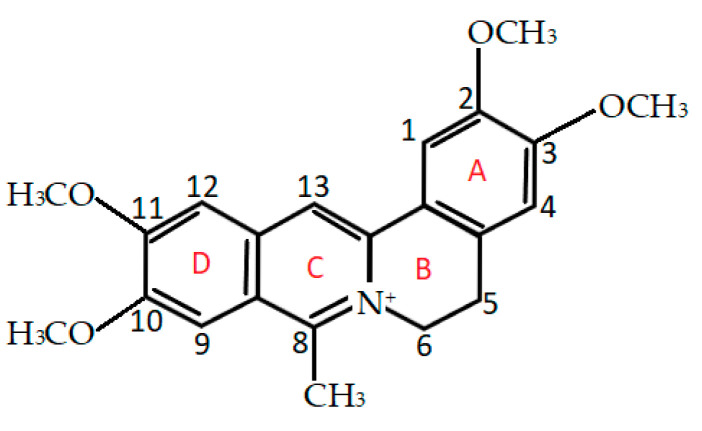
The chemical structure of coralyne.

Isoquinoline-protoberberine alkaloids are an important class of natural metabolites with interesting biological properties; thus, their semi-synthetic and synthetic monomeric and dimeric derivatives have been synthesized. Many studies reported that these compounds have antimicrobial, anti-inflammatory, antimalarial, antiviral, anticholesterol, and anticancer properties [1,2,3]. Whereas the pharmacological effects of naturally occurring protoberberine alkaloids, particularly berberine, have been the subject of extensive in vitro, in vivo and clinical studies, information about the biological properties of coralyne is scarce. Coralyne (13-methyl [1,3] benzodioxolo [5,6-c]-1,3-dioxolo[4, 5-i]-phenanthridium) has the same tetracyclic structure as berberine and palmatine, but differs in terms of its substituents. The chemical structure of coralyne is presented in Figure 1 [4].

Owing to its fully conjugated aromatic system, coralyne has a planar conformation similar to that of berberine [5]. However, the opening of the dioxole ring in the benzodioxole moiety and the presence of an extra methyl group significantly affect its biological activity and DNA binding characteristics, which are different from those reported for the structurally similar berberine [6]. Mechanistic studies revealed that, when bound to proteins, coralyne induced major changes in the proteins’ secondary structure that resulted, among other effects, in strong inhibition of catechol O-methyltransferase, RNA methyltransferases, hemoglobin and lysozyme activities [7]. In vitro experiments demonstrated that coralyne was a potent macrophage activator and had cytostatic properties against various cancer cell lines [8]. Additionally, coralyne formed strong complexes with nucleic acids and inhibited telomerase activity in HeLa cell extract more effectively than berberine, and inhibited topoisomerase I and II activities in cells that are resistant to camptothecin, a well-known topoisomerase I inhibitor [9,10]. The anticancer properties of coralyne against several types of human carcinoma cells were significantly enhanced upon exposure to ultraviolet light, leading to the elicitation of the kinase 2-dependent S phase checkpoint and p53-independent apoptosis. Further research revealed that sensitization of cancer cells to UV light by coralyne, manifested by robust induction of apoptosis, was related to the activation of p38 MAP kinase and the BCL2-associated X protein pathway [11].

Considering the multiplicity of targets and promising anticancer activities of coralyne, it is intriguing to determine whether it increases the sensitivity of cancer cells to ionizing radiation (IR). To investigate this, we analyzed the combined effects of coralyne and IR on cell survival, cell cycle progression, the expression of selected genes and proteins implicated in cell cycle regulation and on the cytoskeleton organization of human lung adenocarcinoma A549 cells in vitro. In addition, the relationship between these effects and cellular uptake of coralyne was studied.

## 2. Results

### 2.1. Kinetics of Coralyne Uptake by A549 Cells

**Figure 2:**
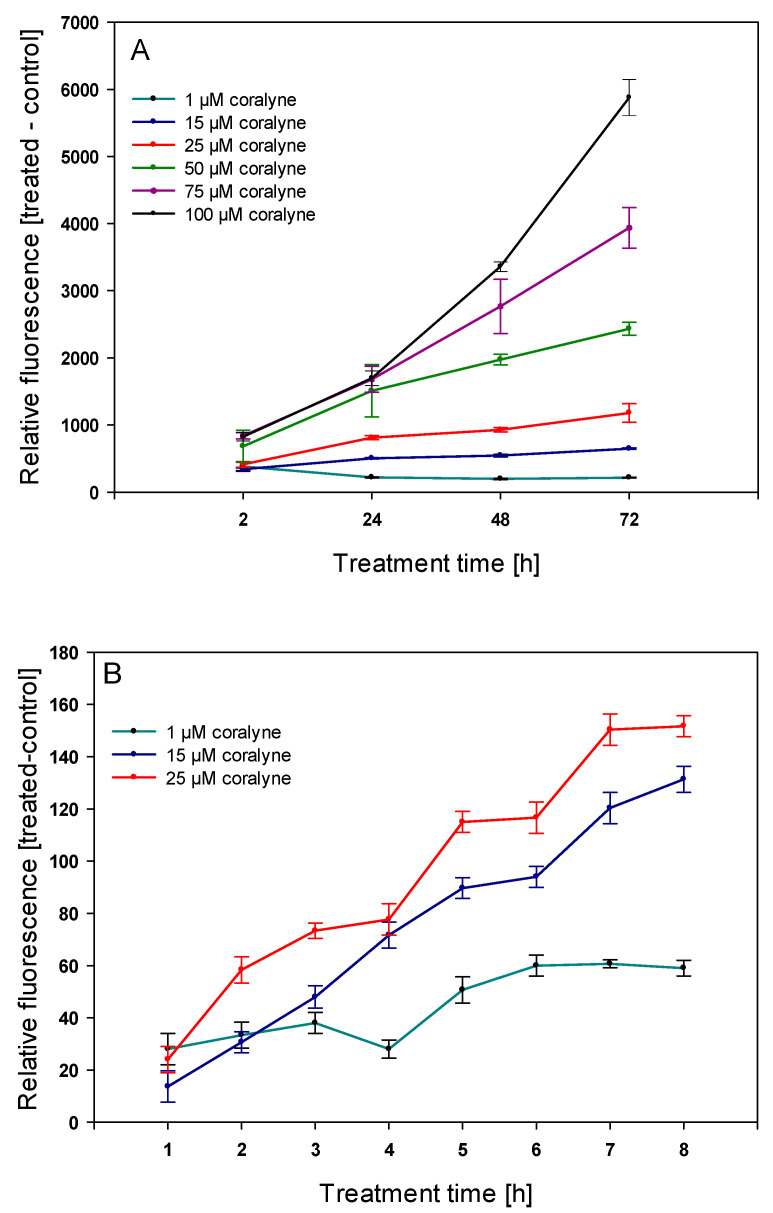
Uptake of coralyne by A549 cells measured by flow cytometry. (**A**) Relative coralyne-associated fluorescence of cells treated with 1–100 µM coralyne for 2, 24, 48, and 72 h. (**B**) Relative coralyne-associated fluorescence of cells treated with coralyne as a function of time. Data represent mean ± SD (*n* = 3).

**Figure 3:**
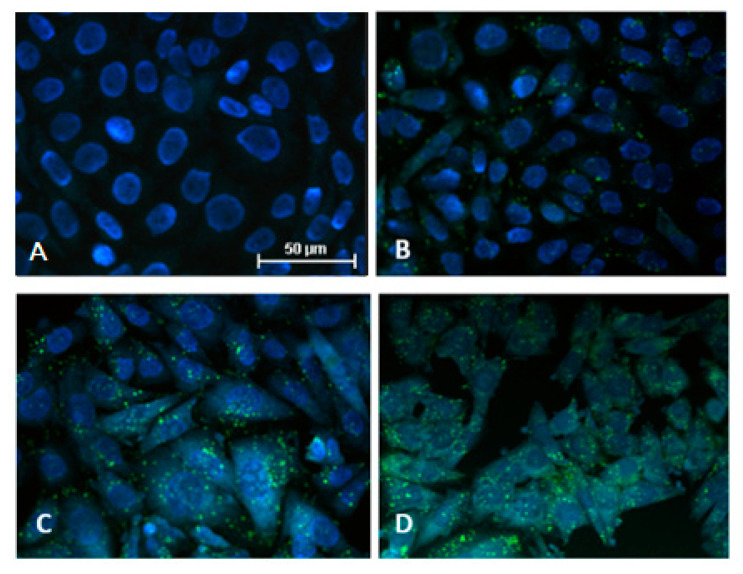
Uptake of coralyne (green) by A549 cells after incubation for 24 h. Nuclei were stained with DAPI (blue). (**A**) Vehicle-treated control cells. (**B**) Cells treated with 1 µM coralyne. (**C**) Cells treated with 25 µM coralyne. (**D**) Cells treated with 50 µM coralyne. Data were recorded and analyzed using the same instrument settings on a laser confocal microscope. Magnification: 40×.

Due to the natural fluorescence of coralyne, we were able to use flow cytometry to measure the kinetics of its uptake by A549 cells. Relative fluorescence of coralyne increased in a dose- and time-dependent manner (Figure 2A,B). It was significantly increased in cells treated with all concentrations of coralyne for 2, 24, 48, and 72 h. These results were confirmed by confocal microscopy, which revealed that coralyne accumulated in the cytoplasm in a dose-dependent manner (Figure 3).

### 2.2. Toxicity of Coralyne in A549 Cells

**Figure 4:**
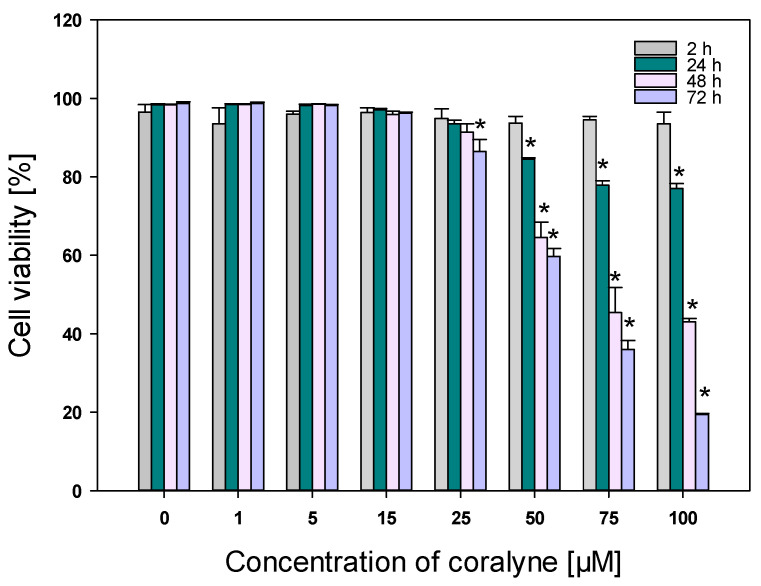
Viability of A549 cells treated with 1–100 µM coralyne for 2, 24, 48, and 72 h. Data represent mean ± SD (*n* = 3). * *p* < 0.05 vs. control cells.

**Figure 5:**
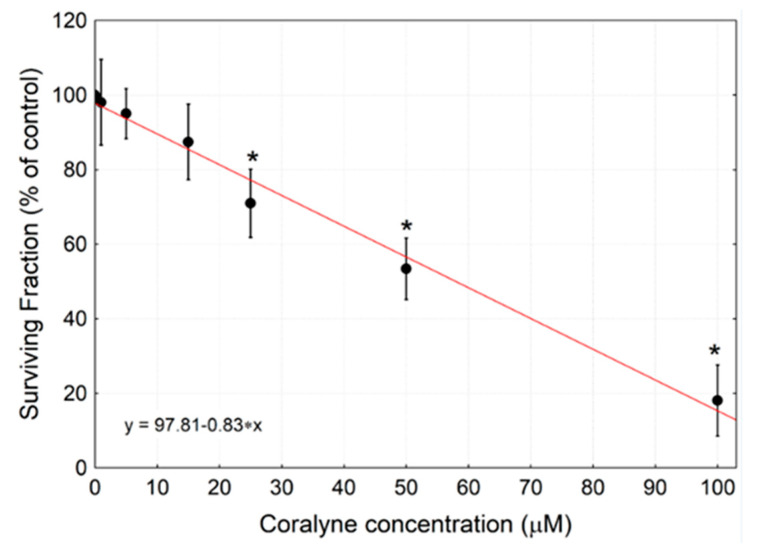
Surviving Fraction of A549 cells exposed to the indicated concentrations of coralyne measured by the clonogenic survival assay. Cells were treated with 1–100 µM coralyne for 24 h, washed and cultured for 10 days. Data are shown as means ± SD (*n* = 3). The trend line is drawn as red line. * *p* < 0.05 vs. control cells.

In the initial set of experiments, the toxicity of coralyne alone was determined. The results of the propidium iodide short-term cytotoxicity assay revealed that continuous treatment of cells with coralyne caused a dose- and time-dependent increase in cell death (Figure 4). The EC50 values for 48 and 72 h were 75.55 and 59.79 µM, respectively. The results of the long-term clonogenic survival assay showed that treatment of the cells with coralyne alone for 24 h caused a dose-dependent inhibition of clonogenic survival (Figure 5). The IC50 and IC75 values were calculated as 45.80 µM and 24.82 µM, respectively. Based on these results, coralyne at the dose of 25 µM was used as the maximum safe dose for further experiments with ionizing radiation to achieve the highest efficacy with minimal side effects.

### 2.3. Coralyne Modulates Radiation-Induced Clonogenic Survival of A549 Cells

**Figure 6:**
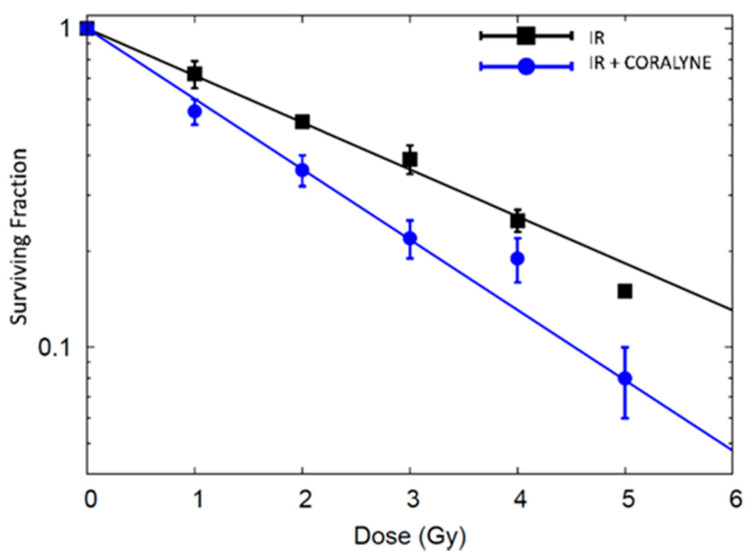
Survival of A549 cells pretreated or not with coralyne (25 µM, 24 h) and exposed to X-rays, as measured by clonogenic survival assay. Data are shown as means ± SD (*n* = 3).

To identify whether coralyne could modulate the sensitivity of cancer cells to ionizing radiation, the survival of cells irradiated with X-rays (1, 2, 3, 4 or 5 Gy) was compared with that of cells pretreated with coralyne (25 µM) and exposed to radiation (Figure 6). The experimental data were fitted with the linear-quadratic model (y = ax + bx^2^ + c). The parameters of the survival curve for cells treated with the combination of coralyne and radiation were 0.592 ± 0.048 Gy −1 and 0.031 ± 0.015 Gy -2 for α and β, respectively. The clonogenic assay showed that the application of coralyne at 25 µM prior to ionizing radiation significantly inhibited the ability of A549 cells to form clones, as compared with irradiated-only cells.

### 2.4. Coralyne Abrogates the G2/M Phase Arrest Caused by Ionizing Radiation in A549 Cells

**Figure 7:**
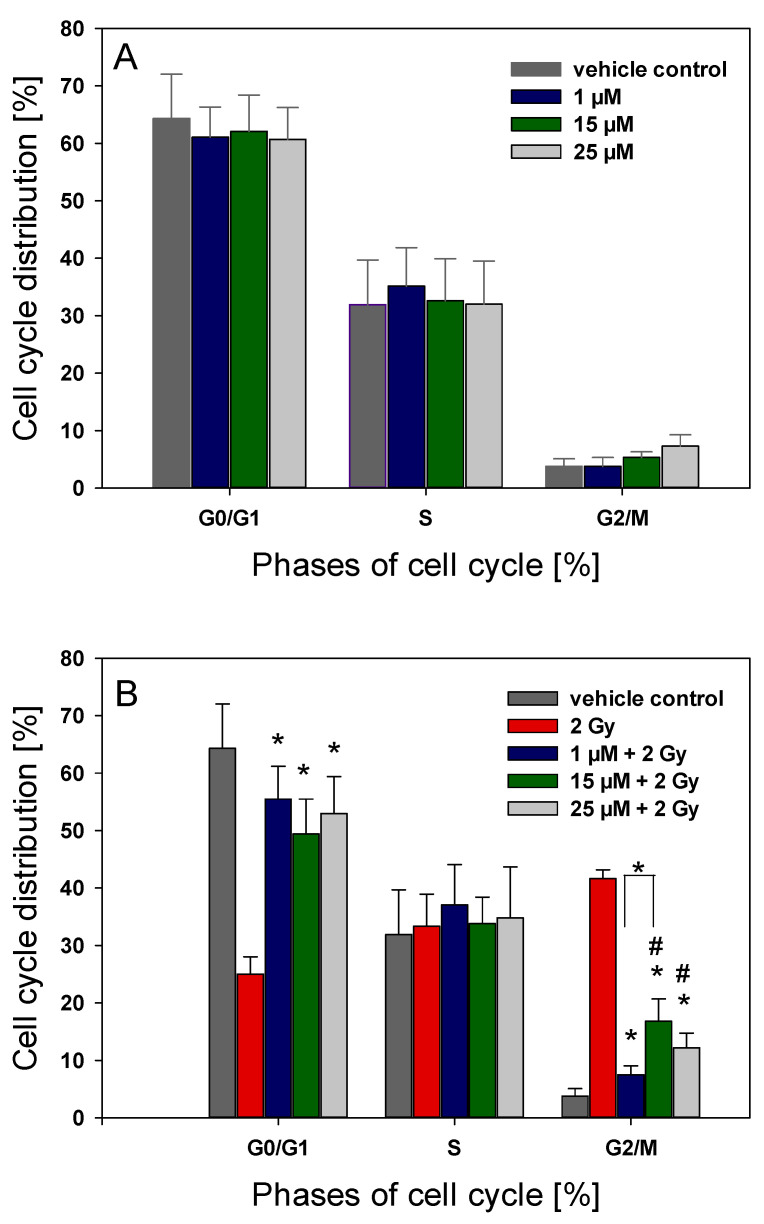
The effect of coralyne on IR-induced changes in the cell cycle distribution of A549 cells. (**A**) The cells were treated with 1–25 µM coralyne for 24 h and harvested after an additional 24 h. The vehicle control was 0.1% DMSO. (**B**) Cells were pretreated with coralyne for 24 h, irradiated with 2 Gy of IR and harvested after an additional 24 h. Data are shown as means ± SD (*n* = 3). * *p* < 0.05 vs. cells treated with 2 Gy of IR alone.; # *p* < 0.05 vs. vehicle control.

Flow cytometry measurements revealed that coralyne did not affect cell cycle distribution (Figure 7A). As expected, ionizing radiation (2 Gy) induced an accumulation of cells in the G2/M phase of the cell cycle (41.63% of the cell population) with a parallel depletion of cells in G0/G1 (3.77%) of the cell population. However, coralyne markedly diminished IR-induced G2/M arrest, which was observed as a shift of cells from G2/M phase into G1 phase. The effect was independent of coralyne concentration (Figure 7B).

### 2.5. Combined Treatment with Coralyne and Ionizing Radiation Up-Regulates the CDKN1A Gene Expression

**Figure 8:**
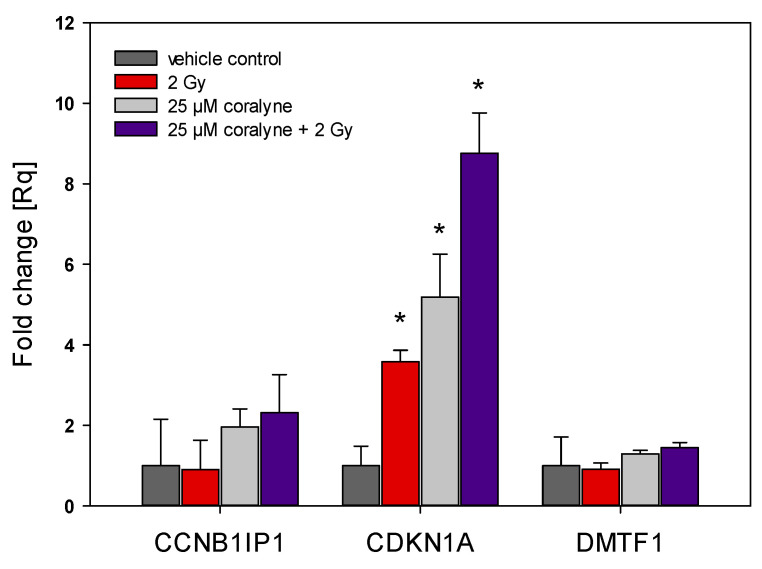
Quantitative real-time RT-PCR analysis of CCNB1IP1, CDKN1A and DMTF1 expression in A549 cells. Data are expressed as mean fold change (Rq) and 0.95 confidence intervals (*n* = 3). * *p* < 0.05 vs. control cells.

To obtain some insights into cell cycle regulation in coralyne and IR treated A549 cells, we analyzed the expression of three genes related to cell cycle progression in cells, namely cyclin B1-interacting protein 1 (CCNB1IP1), cyclin-dependent kinase inhibitor 1 (CDKN1A) and cyclin D-binding Myb-like transcription factor 1 (DMTF1). Treatment with coralyne or IR alone, and in combination, significantly upregulated CDKN1A expression. Combined treatment with coralyne and IR had a synergistic effect on CDKN1A expression (Figure 8). By contrast, the expression of CCNB1IP1 and DMTF1 was not significantly affected.

### 2.6. Effect of Coralyne and IR on Protein Expression

**Figure 9:**
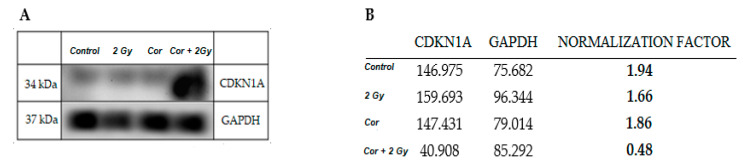
Western blot images (**A**) and densitometric analysis (**B**) of CDKN1A expression in A549 cells (Cor = Coralyne). The densitometric analysis was conducted using imageJ software; the values are presented in 256 gray scale, from 0 (black) to 255 (white). GAPDH was used for normalization of CDKN1A.

**Figure 10:**
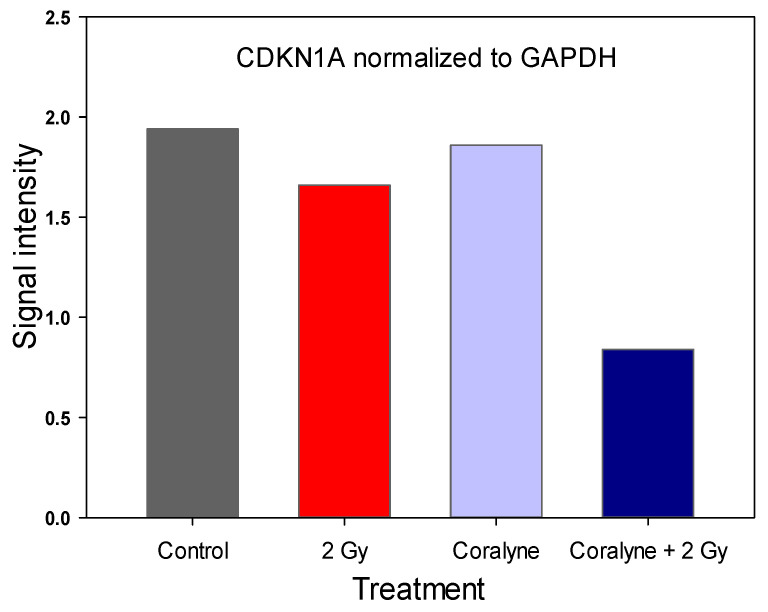
Effect of Coralyne on IR-induced expression of CDKN1A. Signal intensities of CDKN1A from A549 cells were normalized to GAPDH.

Since treatment with coralyne, IR or their combination markedly upregulated *CDKN1A* expression, we decided to assess the expression of the CDKN1A protein, also known as *p21/Cip1*, which is essential for G1/S and G2/M phase transitions of the eukaryotic cell cycle. The level of CDKN1A was the highest in cells treated with 25 µM of coralyne and 2 Gy of IR (Figure 9 and Figure 10).

### 2.7. Effects of Coralyne and IR on the Cytoskeleton Organization

**Figure 11:**
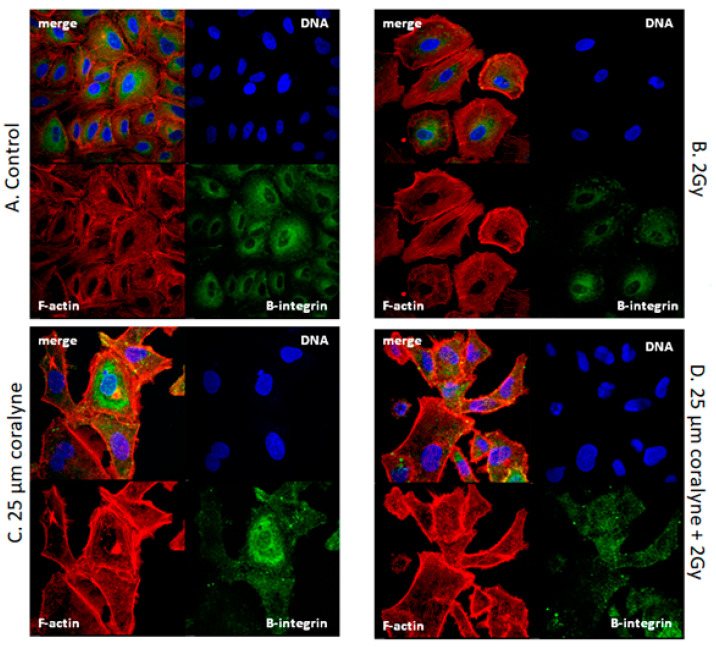
Microscopic visualization of F-actin and integrin-β1 in A549 cells treated with coralyne and exposed to IR. (**A**) Cells treated with vehicle control (0.1% DMSO). (**B**) Cells exposed to 2 Gy of IR. (**C**) Cells treated with 25 μM coralyne for 24 h. (**D**) Cells pretreated with 25 μM coralyne for 24 h and exposed to 2 Gy of IR. Cells were labelled with phalloidin (F-actin, red), FITC (integrin-β1, green), and DAPI (nuclei, blue) and imaged using the same instrument settings on a confocal microscope.

To determine the effects of coralyne and ionizing radiation on the cytoskeleton, we visualized F-actin by direct phalloidin staining. Confocal microscopy analysis showed that, in the control group, F-actin fibers formed a random meshwork, which was distributed throughout the cell (Figure 11A). In irradiated cells, the centrally located F-actin meshwork disappeared and formed serially lined, thin stress fibers in the cytoplasm (Figure 11B). In contrast, cells treated with coralyne alone or with coralyne in combination with ionizing radiation showed a drastic decrease in actin-stress fiber formation, along with altered localization of these fibers towards the cell periphery (Figure 11C,D).

As we observed that coralyne alone as well as coralyne in combination with IR-induced changes in cell spreading and significantly increased attachment of cells to cell culture dishes (data not shown), we visualized β1-integrin by indirect immunofluorescence using β1-integrin specific antibody. Our results revealed that β1 integrins from the control group were distributed throughout the cytoplasm, with some concentration in a perinuclear compartment and with small follicular structures, which were distributed randomly within the plasma (Figure 11A). In irradiated cells, β1 integrins were not perturbed (Figure 11B). In cells treated with coralyne alone or with coralyne in combination with ionizing radiation, β1 integrins accumulated in large cytoplasmic structures (dot-like spots), which were largely distributed around the perimeter of the cell, indicating their translocation (Figure 11C,D).

## 3. Discussion

This study aimed to evaluate the radiosensitizing effect of coralyne in human lung adenocarcinoma A549 cells in vitro and investigate the possible mechanisms responsible for these effects. At first, we measured the kinetics of uptake of coralyne into A549 cells. The flow cytometry measurements were based on the natural fluorescence of coralyne, which is an ionic fluorophore with the absorbance peaks of 219, 231, 300, 311, 326, 360, 405 and 424 nm and an emission spectrum with a maximum 470 nm [12,13]. Moreover, the absorbance and fluorescence spectra of coralyne were previously shown to be unaltered at a pH of 3.0–13.0 and temperatures in the range of 20–70 °C, indicating its usefulness in biological applications under various environmental conditions [12,13]. Our results revealed that coralyne was uptaken by cells in a time- and dose-dependent manner. These results were further confirmed by the confocal microscopy analysis, which showed that coralyne was accumulated in cytoplasmic vacuoles. To the best of our knowledge, the uptake of coralyne into human and animal cells in vitro has not been evaluated, but our results support previous research on berberine, which showed a concentration-, temperature- and time-dependent uptake and accumulation in cellular vacuoles and mitochondria [14,15].

Next, we determined the maximum non-toxic dose of coralyne on A549 cells using the propidium iodide short-term cytotoxicity assay and the long-term survival assay. Our results showed no signs of significant toxicity when the coralyne was permanently available at concentrations ranging from 1 to 15  µM for up to 72 h. When the coralyne was washed from the medium after 24 h, the IC75 and IC50 values from the clonogenic survival assay after 10 days were calculated as ~ 25 µM and 50 µM, respectively. These results are in agreement with the earlier documented in vitro study using coralyne [16]. The authors reported that metabolic activity of A549, MCF7, A431 and MDA-MB-231 was not decreased by the coralyne at concentrations ranging from 0.01 µM to 20 µM after 72 h. In line with these results, Bhattacharyya et al. [11] found that the coralyne at concentrations ranging from 1 µM to 5 µM did not induce any cytotoxicity against U2-OS, A549 and A431 cancer cells after 72 h.

Knowing that 25 µM of coralyne had a significant effect on the clonogenic survival of A549 cells in our study, we applied this dose to investigate the combined effect of coralyne with ionizing radiation. Our results demonstrated that the pretreatment of cells with coralyne prior to X-irradiation effectively enhanced the radiosensitivity of A549 cells. The mechanisms underlying this effect of coralyne have not yet been elucidated. However, Patro et al. [16] and Bhattacharyya et al. [17] reported that the photosensitizing effect of coralyne to ultraviolet A radiation, observed in several human carcinoma cell lines (A549, MCF7, A431 and MDA-MB-231), was associated with the increase in DNA double strand breaks and activation of the ATR-p38 MAPK-BAX pathway. These studies also established that another pathway activated BAX expression in a DNA damage-independent manner, leading to mitochondrial dysfunction and cell death. In addition, a few reports showed that another izoquinoline alkaloid, berberine, sensitized cancer cells to ionizing radiation through the inhibition of DNA repair and the perturbation of cell cycle progression [18,19,20,21,22]. With this in mind, we investigated the effect of coralyne and ionizing radiation on the cell cycle, because it is well known that cell cycle progression significantly affects the radiosensitivity of cancer cells [23]. After DNA damage, cell cycle checkpoints at the G1/S and G2/M transitions are activated to allow time for cells to repair DNA damage before mitosis in order to protect genomic stability [24]. Abrogation of G2/M arrest significantly increases the radiosensitivity of cancer cells [25,26,27].

Our results showed that treatment of cells with a radiosensitizing concentration of 25 µM of coralyne alone did not affect the cell cycle progression. As expected, exposure to 2 Gy of ionizing radiation alone resulted in an enhanced accumulation of A549 cells in the G2/M phase [28,29]. Surprisingly, this effect was significantly reduced when cells were pretreated with 25 µM coralyne for 24 h before irradiation with 2 Gy, suggesting that coralyne can eliminate the radiation-induced G2/M arrest. This confirms that the abrogation of the G2/M arrest effectively increases tumor radiosensitivity [30,31]; thus, coralyne-induced attenuation of ionizing radiation-induced G2/M arrest seems to be a foundation of its radiosensitizing effect. Our results are in line with previous findings that other alkaloids, such as liriodenine and berberine, radiosensitized cells through the abolition of G2/M cell cycle block arrest in combination with ionizing radiation [18,20,32]. Conversely, Patro et al. [16] reported that the combined treatment of A549 cells with coralyne and ultraviolet radiation (C-UVA) resulted in the accumulation of cells in the S-phase.

To further understand the mechanisms behind the observed G2/M phase abrogation and the G1 phase arrest phenotype due to the combined treatment of cells, we investigated the expression of three genes related to cell cycle progression in cells, namely cyclin B1-interacting protein 1 (CCNB1IP1), cyclin-dependent kinase inhibitor 1 (CDKN1A) and cyclin D-binding Myb-like transcription factor 1 (DMTF1). It could be expected that the abrogation of radiation-induced cell cycle arrest by coralyne treatment will be accompanied by a decrease in CDKN1A expression. Surprisingly, we have observed that the combined treatment of A549 cells with coralyne and IR strongly upregulated CDKN1A expression. Treatment of cells with coralyne or IR alone also upregulated CDKN1A expression, but the observed changes were less evident. In contrast, the CCNB1IP1 and DMTF1 mRNA levels were not significantly affected. Based on these results, we assessed the expression of CDKN1A protein, which is essential for the G1/S and G2/M phase transitions of the eukaryotic cell cycle. The level of CDKN1A protein was significantly increased only in cells treated with 25 µM coralyne and 2 Gy of IR. However, the protein level of CDKN1A was not changed in cells exposed to coralyne or IR alone, despite the elevated CDKN1A mRNA levels. In our opinion, the observed difference between the RT-PCR results and the Western blot results is due to different sensitivity of both methods and the anti-CDKN1A antibodies used. Referring to the unexpected interplay between coralyne, CDKN1A and the cell cycle, this complex relationship requires further investigation. It is well known that cells use molecular modulators of cell proliferation, called cyclin-dependent kinase inhibitors (CDKN), to tightly control the cell cycle progression, [33,34]. P21 (CDKN1A) is activated by transcriptional factor p53, whose expression and activation are triggered by DNA damage [33,34]. However, to date, there is no available information about the effects of combined treatment of cells with protoberberines and IR or UV in the context of CDKN1 regulation. It was only reported that berberine—a natural alkaloid of izoquinoline—increased expression of CDKN1A in A431 cells [35]. Furthermore, Qing et al. [36] showed that the expression of CDKN1A is dependent on the concentration of berberine in U266 cells. Other studies [35,37,38] reported that berberine-induced G1 cell cycle arrest is mediated through the increased expression of CDKN proteins (CDKN1A).

Among the factors that can affect cell cycle progression, cytoskeletal organization is considered to be of much relevance. The cytoskeleton filaments are involved in the regulation of cell shape, and either actin filaments or microtubules affect the formation and distribution of cell adhesion. The actin cytoskeleton of cells undergoes drastic changes and remodelling during cell division; in particular, the most dramatic changes occur during the passage through mitosis. In order to analyze the relationship between cytoskeletal organization, the observed G1 cell cycle arrest and G2/M cell cycle abrogation in cells after coralyne and IR treatment, we focused on F-actin and integrin-β1, which were previously shown to be spatially and structurally reorganized in response to radiation in A549 lung cancer cells [39]. Moreover, integrin-mediated cytoskeletal tension is required for cell-cycle progression during the G1 phase. Our results revealed that, in irradiated cells, the F-actin formed thin stress fibers in the cytoplasm. In contrast, cells treated with coralyne alone or with coralyne in combination with ionizing radiation showed a drastic decrease in F-actin stress fiber formation, along with altered localization of these fibers towards the cell periphery. Moreover, we observed reorganization of the integrin-β1 structures in cells treated with coralyne alone and in cells that were pretreated with coralyne and irradiated. Instead of small follicular structures, distributed randomly within the plasma, as in IR-treated cells, we observed large cytoplasmic structures (dot-like spots), which were largely distributed around the perimeter of the cells. As these integrin-β1 spots were not connected with F-actin stress fibers, they seem to be reassembled “focal adhesions” (small adhesions found in membrane protrusions of spreading and migrating cells) [40]. Taken together, the cytoskeleton reorganization observed in cells that were pretreated with coralyne and irradiated may, at least partially, contribute to the observed G1 cell cycle arrest and G2/M cell cycle abrogation. The integrin-mediated increase in substrate attachment in the absence of stress fibers, which regulate cell attachment, migration and morphogenesis, may affect the proper spread and motility of coralyne-pretreated cells, making them more prone to stress. There are a few reports dealing with the influence of protoberberines on cytoskeletal architecture, but none of them deal with IR or UV radiation. Yu et al. [41] showed that treatment of cells with berberine markedly altered the F-actin cytoskeletal architecture. In addition, Hałas et al. [42] reported that caffeine, a purine alkaloid, induced non-small cell lung cancer H1299 cells to acquire a morphology consistent with mitotic catastrophe, with the F-actin network exhibiting diffuse cytoplasmic labelling, characterized by intensely labelled small aggregates of cytoplasmic F-actin that correspond with changes in cell shape. Future studies focused on the effects of coralyne on the pathways responsible for F-actin polymerization and remodelling may provide more mechanistic insights into the effects of coralyne on cell spread alternations and its relevance to radiosensitising effects.

## 4. Materials and Methods

### 4.1. Cell Culture

The A549 human alveolar epithelial cell line was purchased from the American Type Tissue Culture Collection (Rockville, MD, USA). These cells were cultured in F12 Ham medium (Sigma Aldrich, St. Louis, MO, USA) supplemented with 10% fetal bovine serum (Gibco, Thermo Fisher Scientific, Waltham, MA, USA) and 1% penicillin/streptomycin (Gibco, Thermo Fisher Scientific, Waltham, MA, USA) in an atmosphere containing 5% CO_2_ at 37 °C. Cells were passaged by trypsinization using standard methods.

### 4.2. Exposure of Cells to Coralyne and IR

**Figure 12:**
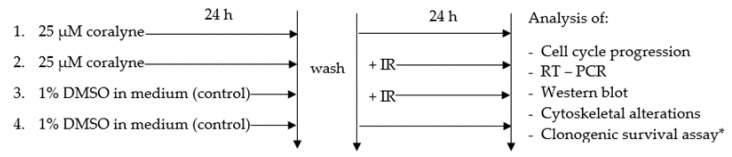
Scheme of the experimental procedure. Four experimental groups were used in this study, namely, cells treated with 25 µM coralyne for 24 h and harvested after a further 24 h, cells treated with 25 µM coralyne for 24 h prior to irradiation and harvested after a further 24 h, cells exposed to IR and harvested after 24 h, and cells treated with the vehicle control. * Cells were incubated for 12 days.

Coralyne chloride (Sigma Aldrich, 99,9%, Steinheim, Germany) was dissolved in dimethyl sulfoxide (DMSO; Sigma-Aldrich, Germany) to obtain appropriate stock solutions and diluted with F12 Ham cell culture medium prior to use (final concentration of DMSO: 0.1%). Two sets of experiments were performed. In the first set, cells were treated with 1–100 µM coralyne for 1–72 h, depending on the endpoints. In the second set, cells were treated with 1–25 µM coralyne in combination with IR. Cells were irradiated with 1–5 Gy of X-rays at a dose rate of 3 Gy/min at the Swietokrzyskie Oncology Center (Kielce, Poland) using the following parameters: radiation field size in isocenter, 30 × 30 cm; SSD distance (source of radiation: phantom surface), 100 cm; depth at which the dose was calculated, 1.4 cm; and radiation energy, 6 MV (Medical Linear Accelerator Artiste, Siemens). Control cells were treated with 0.1% DMSO (Figure 12).

### 4.3. Measurement of Coralyne Uptake by Flow Cytometry

The kinetics of coralyne uptake by A549 cells were examined using a LSR II flow cytometer (Becton Dickinson, Franklin Lakes, NJ, USA). Two sets of experiments were performed. In the first set, cells were treated with 1, 15, 25, 50, 75, and 100 µM coralyne or 0.1% DMSO (vehicle control) for 2, 24, 48 and 72 h. In the second set, cells were treated with 1, 15, and 25 µM coralyne or 0.1% DMSO (vehicle control), and the kinetics of coralyne uptake were measured every hour from 1 to 8 h. After incubation, cells were washed three times with phosphate-buffered saline (PBS), harvested by trypsinization, centrifuged at 1500× *g* for 10 min, and resuspended in PBS (Sigma-Aldrich, St. Louis, MO, USA). Specimens were excited using laser beams with wavelengths of 405 nm and the mean fluorescence emission of coralyne was detected at 480 nm with 20,000 live cells per sample.

### 4.4. Evaluation of Cellular Localization of Coralyne by Confocal Microscopy

Cells were seeded onto sterile round coverslips (Thermo Scientific, Waltham, MA, USA) located in Petri dishes and treated with 1–50 µM coralyne or 0.1% DMSO (vehicle control) for 24 h. Thereafter, the medium was aspirated and cells were washed three times with PBS, fixed in PBS (Sigma-Aldrich, St. Louis, MO, USA) containing 4% paraformaldehyde for 10 min, and washed three times with PBS. Thereafter, coverslips with fixed cells were placed on microscope slides (Medlab Marienfeld, Germany). Cells were analyzed using an A1R confocal microscope (Nikon, Tokyo, Japan). Specimens were excited using laser beams with wavelengths of 405, and then the fluorescence emission of coralyne was measured at 480 nm. However, the fluorescence of DAPI, which was applied for visualisation of cell nuclei, was detected via the UV channel (355 nm excitation and 450 nm emission). Images were acquired with a PlanAPO 10× DIC-L lens. The resolution of the DU4 12-bit photomultiplier tube was set to X:1024 pixels and Y:1024 pixels. The resolving power of the microscope was 0.62 μm/pixel. Image acquisition was obtained within the range of 85 μm. All images were obtained using identical settings to compare fluorescence intensities and cell morphology.

### 4.5. Propidium Iodide (PI) Staining

Cells were incubated with 1–100 µM coralyne or 0.1% DMSO (vehicle control) for 2, 24, 48, and 72 h, washed three times with PBS, harvested by trypsinization, and centrifuged. The pellet was resuspended in 1× binding buffer (50 mM HEPES, 700 mM NaCl, 12.5 mM and CaCl2, pH 7.4) (Becton Dickinson, Franklin Lakes, NJ, USA).) at a density of 1 × 10^6^ cells/mL. The cell suspension (100 µL) was incubated with 5 µL of PI at room temperature for 15 min in the dark. Fluorescence was evaluated using a LSR II flow cytometer (Becton Dickinson, USA). Data were acquired and analyzed using BD FACS DiVa (v 6.0, Becton Dickinson, Franklin Lakes, NJ, USA). Data of 20,000 events were stored.

### 4.6. Clonogenic Assay

To assess clonogenic survival, two sets of experiments were performed. In the first set, cells were treated with 1–100 µM coralyne for 24 h, washed, and trypsinized. An appropriate number of cells was seeded in triplicate into 60 mm Petri dishes in complete medium and incubated for 12 days. In the second set, cells were treated with 25 µM coralyne and exposed to IR according to the scheme of the experimental procedure (Figure 12) with modification of the treatment duration. Briefly, after treatment for 24 h with coralyne and irradiation, cells were washed and trypsinized. An appropriate number of cells was seeded in triplicate into 60 mm Petri dishes in complete medium. Cells were incubated for 12 days and then fixed with methanol (Chempur, Piekary Śląskie, Poland) for 5 min. Colonies were stained with 10% Giemsa solution (Sigma-Aldrich, St. Louis, MO, USA) for 15 min and counted by eye. The plating efficiency was calculated by dividing the number of colonies formed by the number of cells seeded in the control dish. The SF was calculated by dividing the plating efficiency of treated cells by that of untreated cells.

### 4.7. Cell Cycle Analysis

Cells were treated with 1, 15 and 25 µM and exposed to IR according to the scheme of the experimental procedure (Figure 11) and harvested by trypsinization. The cell suspension was centrifuged at 1500× *g* for 10 min and the cell culture medium was discarded. The cell pellet was fixed with 1 mL of cold 80% ethanol at 4 °C for 30 min. Ethanol was removed by centrifugation at 2000 rpm for 5 min and PBS was added to wash the pellets. Cellular DNA was stained with PI solution (50 mg/mL in PBS containing 0.1% Triton X-100, 0.1 mmol/L EDTA, and 50 mg/mL RNase A) for 30 min at room temperature and stored at 4 °C until analysis. DNA was analyzed on a LSR II flow cytometer (Becton Dickinson, USA). Data were acquired with BD FACSDiVa (v 6.0, Becton Dickinson, Franklin Lakes, NJ, USA) and analyzed with Modfit LT software (Verity Software House, Topsham, USA). The percentages of cells in G0/G1, S, and G2/M phases were determined, followed by filtering for doublets and aggregates.

### 4.8. RT-PCR Analysis

Cells were treated with 25 µM coralyne and exposed to IR according to the scheme of the experimental procedure (Figure 11). Total RNA was extracted from cell pellets using an RNeasy Mini Kit (Qiagen, Hilden, Germany Qiagen, US) according to the manufacturer’s protocol. To assess the concentration and purity of RNA, a portion of every RNA sample was diluted with TE buffer (pH 8.0), and absorbance at 230, 260, and 280 nm was measured using an Infinite M200 spectrophotometer (Tecan, Switzerland). All RNA samples used in subsequent analyses had a concentration of ≥100 ng/µL and A260/A280 and A260/A230 ratios of ≥2.0. RNA integrity was tested by agarose gel electrophoresis. For individual gene expression analysis, 1 µg of total RNA was converted to cDNA in a 20 µL reaction volume using a High Capacity cDNA Reverse Transcription Kit (Applied Biosystems, Thermo Fisher Scientific, Waltham, MA, USA) following the manufacturer’s instructions. Thereafter, cDNA was diluted to a volume of 150 µL with deionized nuclease-free H_2_O. Real-time PCR was performed in a 20 µL reaction mixture containing 5 µL of diluted cDNA, 4 µL of deionized nuclease-free H_2_O, 10 µL of TaqMan Universal Master Mix II no UNG (Applied Biosystems, Thermo Fisher Scientific, Waltham, MA, USA), and 1 µL of TaqMan Gene Expression Assay (Applied Biosystems, Thermo Fisher Scientific, Waltham, MA, USA). The following TaqMan assays were used: Hs00355782_m1 (CDKN1A), Hs01565448_g1 (CCNB1IP1), Hs01037749_m1 (DMTF1) and Hs99999905_m1 (GAPDH). All reactions were run in duplicate. PCR amplification was performed using a 7500 Real-Time PCR System (Applied Biosystems, Thermo Fisher Scientific, Waltham, MA, USA) with an initial denaturation step for 10 min at 95 °C followed by 40 cycles of 95 °C for 15 s and 60 °C for 1 min. Relative gene expression was calculated using the ΔΔCt method with GAPDH as a reference control.

### 4.9. Western Blot Analysis

Cells were treated with 25 µM coralyne and exposed to IR according to the scheme of the experimental procedures (Figure 12). Thereafter, cells were washed twice with ice-cold PBS and lysed in radioimmunoprecipitation assay lysis buffer (25 mM Tris-HCl pH 7.6, 150 mM NaCl, 1% NP-40, 1% sodium deoxycholate, and 0.1% SDS) (Sigma-Aldrich, USA) on ice. The lysates were centrifuged for 10 min at 12,000× *g* at 4 °C and the protein concentration was determined with a BCA assay using a Nanodrop 2000 spectrophotometer (Thermo Scientific). Equal amounts of proteins were separated on 12% sodium dodecyl sulfate polyacrylamide gel electrophoresis gels and electrophoretically transferred to nitrocellulose membranes. The membranes were blocked with Western Blocking Buffer (Bio-Rad, UK) at room temperature for 1 h, incubated with anti-CDKN1A and anti-GADPH primary monoclonal antibodies (1 µg/mL; Thermo Fisher Scientific, Rockford, IL, USA) overnight at 4 °C, and then incubated with a horseradish peroxidase-conjugated secondary rabbit anti-mouse antibody (1:5000, Thermo Fisher Scientific) for 1 h. Signals were visualized using Clarity Max Western ECL Blotting Substrates (Bio-Rad), according to the manufacturer’s protocol, and recorded. The densitometric analysis was conducted using imageJ software [43]. The values are presented in 256 gray scale, from 0 (black) to 255 (white).

### 4.10. Analysis of Cytoskeletal Organization

Cells were seeded onto sterile round coverslips coated with poly-L-lysine (Thermo Scientific, US), treated with 25 µM coralyne and exposed to IR according to the scheme of the experimental procedure (Figure 11). Thereafter, cells were fixed using an Image-iT^®^ Fixation/Permeabilization Kit (Life Technologies, US). Briefly, cells were fixed for 15 min with PBS (pH 7.3) containing 4% paraformaldehyde at room temperature, permeabilized with 0.5% Triton X-100 for 15 min, and blocked for 1 h with Dulbecco’s PBS (pH 7.4) containing 3% bovine serum albumin (BSA). The organization of the F-actin network was evaluated in cells stained with rhodamine phalloidin (Invitrogen, US) diluted 1:300 with 0.1% BSA for 20 min at room temperature. The organization of the integrin-β1 network was analyzed in cells stained with an anti-integrin-β1 antibody conjugated with FITC (Abcam, Cambridge, UK) diluted 1:100 with 0.1% BSA for 60 min at room temperature. After staining, cells were washed three times with PBS for 10 min and mounted with SlowFade™ Diamond Antifade Mountant with 4′,6-diamidino-2-phenylindole (DAPI, Life Technologies, Carlsbad, CA, USA) to visualize nuclei. Fluorescent signals were observed with a confocal laser scanning microscope (Nikon A1) equipped with a Nikon Fluor ×60 water immersion objective.

### 4.11. Statistical Analysis

Calculation and statistical analysis of EC50, IC50 and IC75 values were performed using GraphPad Prism 7.0a (GraphPad Software, Inc., San Diego, CA, USA). Clonogenic survival values were normalized to non-treated controls. Survival curves were fitted using the linear-quadratic model S = exp(αD + βD2), where D = radiation dose. Differences between curves were calculated using the extra sum-of-squares F test. Comparisons of cell cycle data were made using Kruskal–Wallis one way analysis of variance on ranks (ANOVA) followed by the post hoc Tukey test. All analysis was performed using Statistica 12 software (Stat Soft. Inc., Tulsa, USA). Data were expressed as mean ± standard deviation (SD) of at least three independent experiments.

## 5. Conclusions

Taken together, our results revealed, for the first time, the radiosensitizing effect of coralyne on human alveolar epithelial cancer A549 cells. The data show that the abrogation of radiation-induced G2/M phase arrest might be the cause of coralyne-dependent radiosensitization and identified an increased expression of cell cycle-related protein CDKN1A as a potential foundation of the effect. However, further experiments are necessary to elaborate the mechanism of coralyne dependence and to confirm the mechanisms of abrogation of radiation-induced G2/M phase arrest in greater detail.

## Data Availability

Not applicable.
